# Reliability of old and new ventricular fibrillation detection algorithms for automated external defibrillators

**DOI:** 10.1186/1475-925X-4-60

**Published:** 2005-10-27

**Authors:** Anton Amann, Robert Tratnig, Karl Unterkofler

**Affiliations:** 1 Innsbruck Medical University, Department of Anesthesia and General Intensive Care, Anichstr. 35, A-6020 Innsbruck, Austria and Department of Environmental Sciences, ETH-Hönggerberg, CH-8093 Zürich, Switzerland; 2 Research Center PPE, FH-Vorarlberg, Achstr. 1, A-6850 Dornbirn, Austria

**Keywords:** ventricular fibrillation detection, automated external defibrillator (AED), ventricular fibrillation (VF), sinus rhythm (SR), ECG analysis

## Abstract

**Background:**

A pivotal component in automated external defibrillators (AEDs) is the detection of ventricular fibrillation by means of appropriate detection algorithms. In scientific literature there exists a wide variety of methods and ideas for handling this task. These algorithms should have a high detection quality, be easily implementable, and work in real time in an AED. Testing of these algorithms should be done by using a large amount of annotated data under equal conditions.

**Methods:**

For our investigation we simulated a continuous analysis by selecting the data in steps of one second without any preselection. We used the complete BIH-MIT arrhythmia database, the CU database, and the files 7001 – 8210 of the AHA database. All algorithms were tested under equal conditions.

**Results:**

For 5 well-known standard and 5 new ventricular fibrillation detection algorithms we calculated the sensitivity, specificity, and the area under their receiver operating characteristic. In addition, two QRS detection algorithms were included. These results are based on approximately 330 000 decisions (per algorithm).

**Conclusion:**

Our values for sensitivity and specificity differ from earlier investigations since we used no preselection. The best algorithm is a new one, presented here for the first time.

## Background

Sudden cardiac arrest is a major public health problem and one of the leading causes of mortality in the western world. In most cases, the mechanism of onset is a ventricular tachycardia that rapidly progresses to ventricular fibrillation [1]. Approximately one third of these patients could survive with the timely employment of a defibrillator.

Besides manual defibrillation by an emergency paramedic, bystander defibrillation with (semi-)automatic external defibrillators (AEDs) has also been recommended for resuscitation. These devices analyze the electrocardiogram (ECG) of the patient and recognize whether a shock should be delivered or not, as, e.g., in case of ventricular fibrillation (VF). It is of vital importance that the ECG analysis system used by AEDs differentiates well between VF and a stable but fast sinus rhythm (SR). An AED should not deliver a shock to a collapsed patient not in cardiac arrest. On the other hand, a successfully defibrillated patient should not be defibrillated again.

The basis of such ECG analysis systems of AEDs is one or several mathematical ECG analysis algorithms. The main purpose of this paper is to compare various old and new algorithms in a standardized way. To gain insight into the quality of an algorithm for ECG analysis, it is essential to test the algorithms under equal conditions with a large amount of data, which has already been commented on by qualified cardiologists.

Commonly used annotated databases are Boston's Beth Israel Hospital and MIT arrhythmia database (BIH-MIT), the Creighton University ventricular tachyarrhythmia database (CU), and the American Heart Association database (AHA).

We used the complete CU and BIH-MIT arrhythmia database, and the files 7001 – 8210 of the AHA database, [2], [3,4]. For each algorithm approximately 330 000 decisions had been calculated. No preselection of certain ECG episodes was made, which mimics the situation of a bystander more accurately. In this investigation we analyzed 5 well-known standard and 5 new ventricular fibrillation detection algorithms. In addition, two QRS detection algorithms were included. The results are expressed in the quality parameters *Sensitivity *and *Specificity*. In addition to these two parameters, we calculated the *Positive Predictivity *and *Accuracy *of the investigated algorithms. Furthermore, the calculation time in comparison to the duration of the real data was calculated for the different algorithms. The calculation times were obtained by analyzing data from the CU database only.

**Figure 1:**
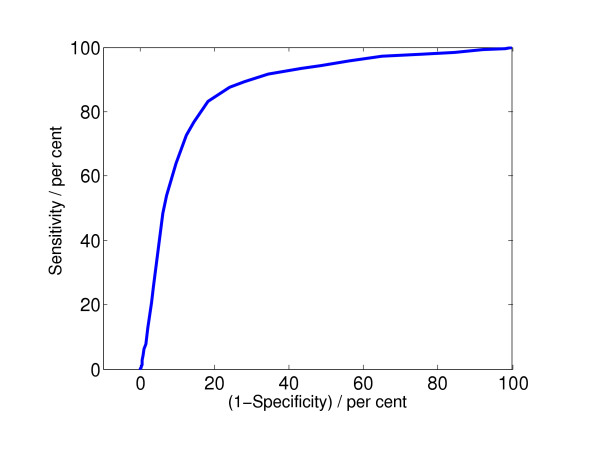
Receiver operating characteristic for the algorithm "complexity measure" described in the introduction, for a window length of 8 s. The calculated value for the area under the curve, IROC, is 0.87.

The quality parameters were obtained by comparing the VF/no VF decisions suggested by the algorithm with the annotated decisions suggested by cardiologists. The cardiologists' decisions are considered to be correct. We distinguished only between ventricular fibrillation and no ventricular fibrillation, since the annotations do not include a differentiation between ventricular fibrillation and ventricular tachycardia. The closer the quality parameters are to 100%, the better the algorithm works. Since an AED has to differentiate between VF and no VF, the sensitivity and specificity are the appropriate parameters. To represent the quality of an algorithm by its sensitivity and specificity bears some problems. A special algorithm can have a high sensitivity, but a low specificity, or conversely. Which one is better? To arrive at a common and single quality parameter, we use the receiver operating characteristic (ROC). The sensitivity is plotted in dependence of (1 - specificity), where different points in the plot are obtained by varying the critical threshold parameter in the decision stage of the algorithm. By calculating the area under the ROC curve (we call this value "integrated receiver operating characteristic", and denote it by IROC), it is possible to compare different algorithms by one single value. Figure 1 shows a typical example of an ROC curve.

**Figure 5:**
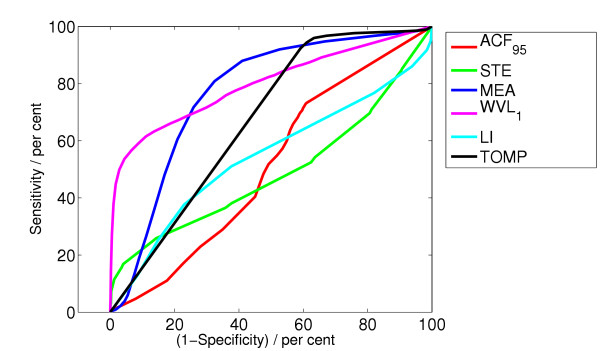
ROC curve for the algorithms ACF, STE, MEA, WVL_1_, LI, TOMP.

**Figure 6:**
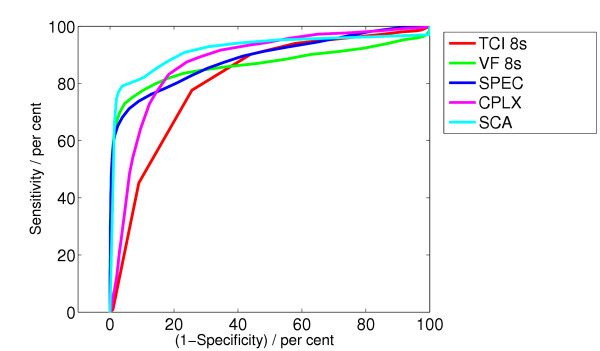
ROC curve for the algorithms TCI, VF, SPEC, CPLX, SCA.

Section 1 provides the necessary background for the algorithms under investigation. Section 2 describes the methods of evaluation and represents our results in Table 1, 2, 3 and Figure 5 and 6. A discussion of the results follows in Section 3. Appendix A recalls the basic definitions of the quality parameters Sensitivity, Specificity, Positive Predictivity, Accuracy, and ROC curve. In Appendix B we provide more details on one of the new algorithms.

**Table 1:** >Quality of ventricular fibrillation detection algorithms (sensitivity (Sns), specificity (Spc), integrated receiver operating characteristic (IROC)) in per cent, rounded on 3 significant digits, wl = window length in seconds. (*) ... no appropriate parameter exists.

*Data Source*		*MIT DB*		*CU DB*		*AHA DB*		*overall result*		
Parameter	wl	Sns.	Spc.	Sns.	Spc.	Sns.	Spc.	Sns.	Spc.	IROC

TCI	3	82.5	78.1	73.5	62.6	75.2	78.3	75.0	77.5	82
TCI	8	74.5	83.9	71.0	70.5	75.7	86.9	75.1	84.4	82
ACF_95_	8	33.2	45.9	38.1	58.9	51.5	52.2	49.6	49.0	49
ACF_99_	8	59.4	30.1	54.7	49.3	71.5	40.3	69.2	35.0	49
VF	4	36.7	100	32.2	99.5	17.6	99.9	19.6	99.9	85
VF	8	29.4	100	30.8	99.5	16.9	100	18.8	100	87
SPEC	8	23.1	100	29.0	99.3	29.2	99.8	29.1	99.9	89
CPLX	8	6.3	92.4	56.4	86.6	60.2	91.9	59.2	92.0	87
STE	8	54.5	83.4	52.9	66.6	49.6	81.0	50.1	81.7	67
MEA	8	62.9	80.8	60.1	87.5	49.8	88.6	51.2	84.1	82
SCA	8	72.4	98.0	67.7	94.9	71.7	99.7	71.2	98.5	92
WVL_1_	8	28.7	99.9	26.2	99.4	26.8	99.5	26.7	99.7	80
WVL_2_	8	81.1	89.0	61.0	72.1	73.5	89.6	72.0	88.4	(*)
LI	8	3.1	95.1	7.5	94.8	9.3	92.0	9.0	93.9	58
TOMP	8	68.5	40.6	71.3	48.4	95.9	39.7	92.5	40.6	67

**Table 2:** Quality of ventricular fibrillation detection algorithms (positive predictivity (PP), accuracy (Ac), calculation time(ct)) for a window length of 8 seconds. Positive predictivity and accuracy in per cent, rounded on 3 digits; calculation time in per cent of the real time of the data, rounded on 2 digits, wl = window length in seconds.

*Data Source*		*MIT DB*		*CU DB*		*AHA DB*		*overall result*		
Parameter	wl	PP.	Ac.	PP.	Ac.	PP.	Ac.	PP.	Ac.	ct.

TCI	3	0.6	78.1	33.9	64.8	41.7	77.8	23.7	77.2	1.5
TCI	8	0.8	83.9	38.9	70.6	54.4	84.9	31.1	83.6	2.1
ACF_95_	8	0.1	45.9	19.7	54.5	18.2	52.1	8.3	49.0	3.6
ACF_99_	8	0.1	30.2	22.2	50.4	19.9	45.7	9.1	37.9	3.6
VF	4	91.3	99.9	94.0	85.5	98.0	85.8	97.0	93.1	1.4
VF	8	82.4	99.9	94.5	85.2	98.9	85.7	97.7	93.0	1.9
SPEC	8	60.6	99.8	92.0	84.6	97.3	87.7	96.1	93.8	1.9
CPLX	8	0.1	92.3	52.7	80.3	60.7	86.5	40.8	89.2	2.5
STE	8	0.5	83.4	29.5	63.8	35.1	75.6	20.4	79.0	1.9
MEA	8	0.5	80.8	56.0	81.8	47.5	81.9	23.2	81.3	2.5
SCA	8	5.6	97.9	77.8	89.2	98.0	94.9	81.6	96.2	5.9
WVL_1_	8	38.9	99.8	92.1	84.1	91.8	87.0	90.5	93.5	1.9
WVL_2_	8	1.2	88.9	36.6	69.8	59.3	86.8	36.8	87.0	40
LI	8	0.1	94.9	27.5	76.5	19.4	77.8	12.1	86.6	15
TOMP	8	0.2	40.6	26.7	53.2	24.8	49.4	12.7	45.0	0.84

**Table 3:** Sensitivity of ventricular fibrillation detection algorithms in per cent, wl = window length in seconds.

Parameter	wl	Sns. if Spc. = 95	Sns. if Spc. = 99
TCI	3	15.0	1.0
TCI	8	25.3	1.3
ACF_95_	8	3.0	0.6
ACF_99_	8	3.0	0.6
VF	4	71.0	59.2
VF	8	73.4	59.7
SPEC	8	69.8	58.9
CPLX	8	38.8	5.8
STE	8	29.4	10.8
MEA	8	7.0	0.5
SCA	8	79.0	66.4
WVL_1_	8	56.7	35.2
LI	8	7.3	1.4
TOMP	8	9.1	1.8

## 1 Methods

The ventricular fibrillation detection algorithms considered here are partly taken from the scientific literature, five of them are new. Some of them have been evaluated in [5] and [6].

For all algorithms we used the same prefiltering process. First, a moving average filter of order 5 is applied to the signal. (Note 1: Fixing the order of 5 seems to bear a problem. Data with different frequencies (AHA, CU ... 250 Hz, BIH-MIT ... 360 Hz) are filtered in a different way. But this is neglectable, when the Butterworth filter is applied afterwards.) This filter removes high frequency noise like interspersions and muscle noise. Then, a drift suppression is applied to the resulting signal. This is done by a high pass filter with a cut off frequency of one Hz. Finally, a low pass Butterworth filter with a limiting frequency of 30 Hz is applied to the signal in order to suppress needless high-frequency information even more. This filtering process is carried out in a MATLAB routine. It uses functions from the "Signal Processing Toolbox". (Note 2: We used MATLAB R13 – R14 and "Signal Processing Toolbox" version 6.1 – 6.3 on a Power Mac G5, 2 GHz.) In order to obtain the ROC curve we have to change a parameter which we call "critical threshold parameter" below.

### TCI algorithm

The threshold crossing intervals algorithm (TCI) [7] operates in the time domain. Decisions are based on the number and position of signal crossings through a certain threshold.

First, the digitized ECG signal is filtered by the procedure mentioned above. Then a binary signal is generated from the preprocessed ECG data according to the position of the signal above or below a given threshold. The threshold value is set to 20% of the maximum value within each one-second segment S and recalculated every second. Subsequent data analysis takes place over successive one-second stages. The ECG signal may cross the detection threshold one or more times, and the number of pulses is counted. For each stage, the threshold crossing interval *TCI *is the average interval between threshold crossings and is calculated as follows



**Figure 2:**
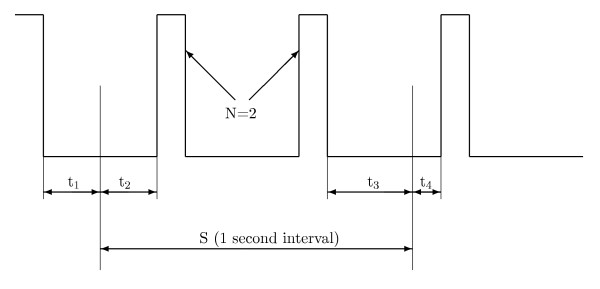
Binary signal with 2 pulses in threshold crossing intervals algorithm.

Figure 2 illustrates the situation.

Here, *N *is the number of pulses in S. *t*_1 _is the time interval from the beginning of S back to the falling edge of the preceding pulse. *t*_2 _is the time interval from the beginning of S to the start of the next pulse. *t*_3 _is the interval between the end of the last pulse and the end of S and *t*_4 _is the time from the end of S to the start of the next pulse.

If *TCI *≥ *TCI*_0 _= 400 *ms*, SR is diagnosed. Otherwise sequential hypothesis testing [7] is used to separate ventricular tachycardia (VT) from VF.

As stated above, the original algorithm works with single one-second time segments, (see [7], page 841). To achieve this the algorithm picks a 3-second episode. The first second and the third second are used to determine *t*_1 _and *t*_4_. The 2nd second yields the value for *TCI*. When picking an 8-second episode we can hence evaluate 6 consecutive *TCI *values. Final SR decision is taken if diagnosed in four or more segments otherwise the signal is classified as VF.

The critical threshold parameter to obtain the ROC is *TCI*_0_.

### ACF algorithm

The autocorrelation algorithms ACF_95 _(Note 3: Probability of 95% in the Fisher distribution → *α *= 0.05 in F(*α*, *k*_1_, *k*_2_) with *k*_1 _= 1, *k*_2 _= 5) and ACF_99 _(Note 4: Probability of 99% in the Fisher distribution → *α *= 0.01 in *F*(*α*, *k*_1_, *k*_2_) with *k*_1 _= 1, *k*_2 _= 5) [8] analyze the periodicities within the ECG. Given a discrete signal *x*(*m*), the short-term autocorrelation function (ACF) of *x*(*m*) with a rectangular window is calculated by



Here, this technique is used to separate VT and SR from VF. It is assumed that VF signals are more or less aperiodic and SR signals are approximately periodic.

This assumption is however questionable since VF signals may have a cosine like shape. Compare this assumption with the assumption made by the algorithm in the next subsection.

Note that the autocorrelation function of a function *f *is connected to the Power Spectrum of  by the "Wiener-Khinchin Theorem".

The detection algorithm performs a linear regression analysis of ACF peaks. An order number *i *is given to each peak according to its amplitude. So, the highest peak is called *P*_0_, etc., ranged by decreasing amplitudes. In a SR signal, which is considered to be periodic or nearly periodic, a linear relationship should exist between the peaks lag and their index number *i*. No such relationship should exist in VF signals. The linear regression equation of the peak order and its corresponding lag of *m *peaks in the ACF is described as

*y*_*i *_= *a *+ *bx*_*i*_,     (3)

where *x*_*i *_is the peak number (from 0 to (*m *- 1)), and *y*_*i *_is the lag of *P*_*i*_.



In this study, *m *= 7. The variance ratio *VR *is defined by



where



If *VR *≥ *VR*_0 _is greater than the Fisher statistics for degrees of freedom *k*_1 _= 1 and *k*_2 _= *m *- 2 = 5 with 95% (99%) probability, the rhythm is classified by ACF_95 _(ACF_99_) to be SR, otherwise it is VF. The critical threshold parameter to obtain the ROC is *VR*_0_, (*VR*_0 _≈ 6.61(16.3) at 95% (99%)).

### VF filter algorithm

The VF filter algorithm (VF) [9] applies a narrow band elimination filter in the region of the mean frequency of the considered ECG signal.

After preprocessing, a narrow band-stop filter is applied to the signal, with central frequency being equivalent to the mean signal frequency *f*_*m*_. Its calculated output is the "VF filter leakage". The VF signal is considered to be approximately of sinusoidal waveform.

The number *N *of data points in an average half period *N *= *T*/2 = 1/(2*f*_*m*_) is given by



where *V*_*i *_are the signal samples, *m *is the number of data points in one mean period, and ⌊...⌋ denotes the floor function. The narrow band-stop filter is simulated by combining the ECG data with a copy of the data shifted by a half period. The VF-filter leakage *l *is computed as



In the original paper [9] this algorithm is invoked only if no QRS complexes or beats are detected. This is done by other methods. Since we employ no prior QRS detection, we use the thresholds suggested by [5]. If the signal is higher than a third of the amplitude of the last by the VF-filter detected QRS complex in a previous segment and the leakage is smaller than *l*_0 _= 0.406, VF is identified. Otherwise the leakage must be smaller than *l*_0 _= 0.625 in order to be classified as VF.

The critical threshold parameter to obtain the ROC is the leakage *l*_0_.

### Spectral algorithm

The spectral algorithm (SPEC) [10] works in the frequency domain and analyses the energy content in different frequency bands by means of Fourier analysis.

The ECG of most normal heart rhythms is a broadband signal with major harmonics up to about 25 *Hz*. During VF, the ECG becomes concentrated in a band of frequencies between 3 and 10 *Hz *(cf. [11,12], with particularly low frequencies of undercooled victims).

After preprocessing, each data segment is multiplied by a Hamming window and then the ECG signal is transformed into the frequency domain by fast Fourier transform (FFT). The amplitude is approximated in accordance with ref. [10] by the sum of the absolute value of the real and imaginary parts of the complex coefficients. (Note 5: Normally one would take the modulus of complex amplitudes.) Let Ω be the frequency of the component with the largest amplitude (called the peak frequency) in the range 0.5 – 9 Hz. Then amplitudes whose value is less than 5% of the amplitude of Ω are set to zero. Four spectrum parameters are calculated, the normalized first spectral moment *M*



*j*_*max *_being the index of the highest investigated frequency, and *A*_1_, *A*_2_, *A*_3_. Here *w*_*j *_denotes the *j*-th frequency in the FFT between 0 Hz and the minimum of (20Ω, 100 Hz) and *a*_*j *_is the corresponding amplitude. *A*_1 _is the sum of amplitudes between 0.5 Hz and Ω/2, divided by the sum of amplitudes between 0.5 Hz and the minimum of (20Ω, 100 Hz). *A*_2 _is the sum of amplitudes between 0.7Ω and 1.4Ω divided by the sum of amplitudes between 0.5 Hz and the minimum of (20Ω, 100 Hz). *A*_3 _is the sum of amplitudes in 0.6 Hz bands around the second to eighth harmonics (2Ω – 8Ω), divided by the sum of amplitudes in the range of 0.5 Hz to the minimum of (20Ω, 100 Hz).

VF is detected if *M *≤ *M*_0 _= 1.55, *A*_1 _<*A*_1,0 _= 0.19, *A*_2 _≥ *A*_2,0 _= 0.45, and *A*_3 _≤ *a*_3,0 _= 0.09.

The critical threshold parameter to obtain the ROC is *A*_2,0_, the other threshold parameters (*A*_1,0_, *A*_3,0_, *M*_0_) being kept constant.

### Complexity measure algorithm

The complexity measure algorithm (CPLX) [13] transforms the ECG signal into a binary sequence and searches for repeating patterns.

Lempel and Ziv [14] have introduced a complexity measure *c*(*n*), which quantitatively characterizes the complexity of a dynamical system.

After preprocessing, a 0 – 1 string is generated by comparing the ECG data *x*_*i *_(*i *= 1...*n*, *n *being the number of data points) to a suitably selected threshold. The mean value *x*_*m *_of the signal in the selected window is calculated. Then *x*_*m *_is subtracted from each signal sample *x*_*i*_. The positive peak value *V*_*p*_, and the negative peak value *V*_*n *_of the data are determined.

By counting, the quantities *P*_*c *_and *N*_*c *_are obtained. *P*_*c *_represents the number of data *x*_*i *_with range 0 <*x*_*i *_< 0.1*V*_*p *_and *N*_*c *_the number of data *x*_*i *_with range 0.1*V*_*n *_<*x*_*i *_< 0. If (*P*_*c *_+ *N*_*c*_) < 0.4*n*, then the threshold is selected as *T*_*d *_= 0. Else, if *P*_*c *_<*N*_*c*_, then *T*_*d *_= 0.2*V*_*p*_, otherwise *T*_*d *_= 0.2*V*_*n*_. Finally, *x*_*i *_is compared with the threshold *T*_*d *_to turn the ECG data into a 0 – 1 string *s*_1_*s*_2_*s*_3 _... *s*_*n*_. If *x*_*i *_<*T*_*d*_, then *s*_*i *_= 0, otherwise *s*_*i *_= 1. Now, from this string a complexity measure *c*(*n*) is calculated by the following method, according to [14].

If *S *and *Q *represent two strings then *SQ *is their concatenation. *SQπ *is the string *SQ *when the last element is deleted. Let *v*(*SQπ*) denote the vocabulary of all different substrings of *SQπ*. At the beginning, *c*(*n*) = 1, *S *= *s*_1_, *Q *= *s*_2_, and therefore *SQπ *= *s*_1_. For generalization, now suppose *S *= *s*_1_*s*_2 _... *s*_*r *_and *Q *= *s*_*r*+1_. If *Q *∊ *v*(*SQπ*), then *s*_*r*+1 _is a substring of *s*_1_*s*_2 _... *s*_*r*_, therefore *S *does not change. *Q *has to be renewed to be *s*_*r*+1_*s*_*r*+2_. Then it has to be judged if *Q *belongs to *v*(*SQπ*) or not. This procedure has to be carried out until *Q *∉ *v*(*SQπ*), now *Q *= *s*_*r*+1 _*s*_*r*+2 _... *s*_*r*+*i*_, which is not a substring of *s*_1_*s*_2 _... *s*_*r*_*s*_*r*+1 _... *s*_*r*+*i*-1_, thus *c*(*n*) is increased by one. Thereafter *S *is combined with *Q*, and *S *is renewed to be *S *= *s*_1_*s*_2 _... *s*_*r*_*s*_*r*+1 _... *s*_*r*+*i*_, and at the same time *Q *has to be renewed to be *Q *= *s*_*r*+*i*+1_. The above procedures are repeated until *Q *contains the last character. At this time the number of different substrings of *s*_1_, *s*_2_, ..., *s*_*n *_is *c*(*n*), i.e., the measure of complexity, which reflects the rate of new pattern arising with the increase of the pattern length *n*.

The normalized *C*(*n*) is computed:



where *b*(*n*) gives the asymptotic behavior of *c*(*n*) for a random string:



Evidently, 0 ≤ *C*(*n*) ≤ 1. In order to obtain results that are independent of *n*, *n *must be larger than 1000. Since *n *is given by window length *wl *times sampling rate, we choose *wl *= 8*s*.

If *C *<*C*_0 _= 0.173 the ECG is classified as SR, if 0.173 ≤ *C *≤ 0.426 as VT, and if *C *> *C*_1 _= 0.426 as VF. A shock is recommended only if *C *> *C*_1_.

The critical threshold parameter to obtain the ROC is *C*_0_.

### Standard exponential algorithm

The standard exponential (STE) algorithm counts the number of crossing points of the ECG signal with an exponential curve decreasing on both sides. The decision for the defibrillation is made by counting the number of crossings. This simple algorithm is probably well-known, but we did not find any description of it in the literature.

**Figure 3:**
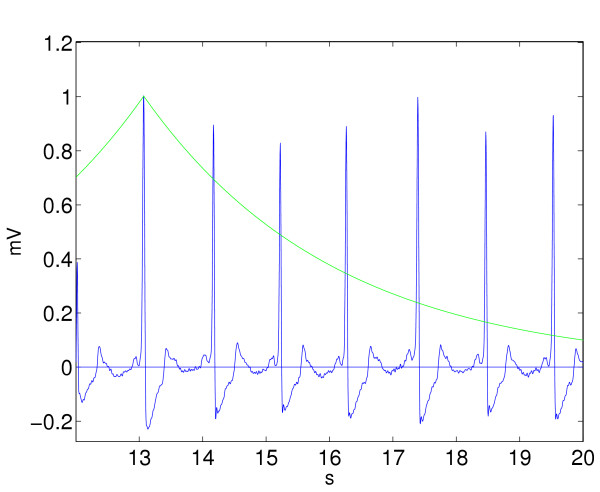
A 8 second episode of SR rhythm is investigated with the standard exponential algorithm (STE). The exponential function intersects the signal 12 times.

The ECG signal is investigated in the time domain. First, the absolute maximum value of the investigated sequence of the signal is searched. An exponential like function *E*_*s*_(*t*) is put through this point. This function is decreasing in both directions. Hence, it has the representation:



Here, *M *is the amplitude of the signal maximum, *t*_*m *_is the corresponding time, *τ *is a time constant. In our investigation, *τ *is set to 3 seconds. The number of intersections *n *of this curve with the ECG signal is counted and a number *N *is calculated by



where *T *is the time length of the investigated signal part. If *N *> *N*_0 _= 250 crossings per minute (cpm), the ECG-signal is classified as VF. If *N *<*N*_1 _= 180 cpm, SR is identified. Otherwise the signal is classified as VT. Figure 3 illustrates the situation (note that each QRS complex intersects the exponential function two times).

A shock is recommended only if *N *> *N*_0_.

The critical threshold parameter to obtain the ROC is *N*_0_.

### Modified exponential algorithm

A modified version of STE, called MEA lifts the decreasing exponential curve at the crossing points onto the following relative maximum. This modification gives rise to better detection results.

This algorithm works in the time domain. First, the first relative maximum value of the investigated sequence of the signal is searched and an exponential like function *E*_*n*,1_(*t*) is put through this point. Here, it has the representation:



with *M*_*j *_being the value of the j-th relative maximum of the signal, *t*_*m*,*j *_the corresponding time and *τ *the time constant. Here, *τ *is set to 0.2 seconds. *t*_*c*,*j *_is the time value, where the exponential function crosses the ECG signal.

The difference to STE is, that here the function does not have the above representation over the whole investigated signal part, but only in the region from the first relative maximum to the first intersection with the ECG signal. Then, the function *E*_*n*,*j*_(*t*) coincides with the ECG signal until it reaches a new relative maximum. In some way one can say that the function *MEA*(*t*) is "lifted" here from a lower value to a peak. From that peak on it has again the above representation with *M *being the value of the next relative maximum. This is done until the curve reaches the end of the investigated ECG sequence.

The number of the liftings *n *of this curve with the ECG signal is counted and a number *N *is calculated by



where *T *is the time length of the investigated signal part. If *N *> *N*_0 _= 230 crossings per minute (cpm), the ECG-signal is classified as VF. If *N *<*N*_1 _= 180 cpm, SR is identified.

**Figure 4:**
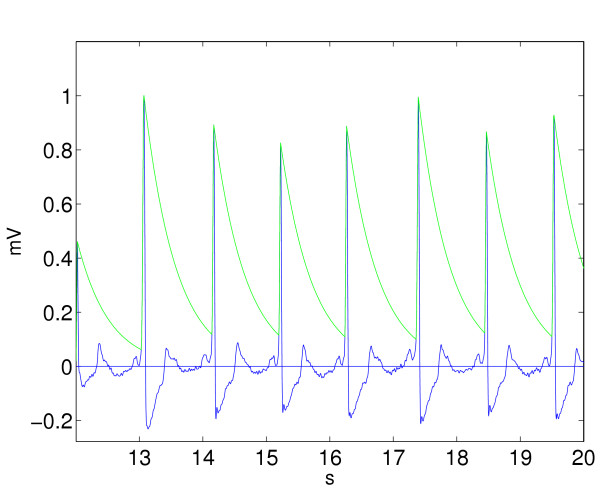
A 8 second episode of SR rhythm is investigated with the modified exponential algorithm (MEA). The exponential function is lifted 7 times.

Otherwise the signal is classified as VT. Figure 4 illustrates the situation. A shock is recommended only if *N *> *N*_0_.

The critical threshold parameter to obtain the ROC is *N*_0_.

### Signal comparison algorithm

This new algorithm (SCA) compares the ECG with four predefined reference signals (three sinus rhythms containing one PQRST segment and one ventricular fibrillation signal) and makes its decision by calculation of the residuals in the L^1 ^norm.

The algorithm works in the time domain. After preprocessing, all relative maxima of a modified ECG signal are searched. The relative positions in time *t*_*j *_and amplitude *a*_*j *_of these points are considered. We call this set *M*_0_, with *M*_0 _= {(*t*_*j*_, *a*_*j*_)|*a*_*j *_is a local maximum}. With this information a probability test for being the peak of a possible QRS complex is performed. For a detailed description of this test see steps 1 and 2 in Appendix B. In a normal ECG, most of the relative maxima *M*_0 _of the ECG signal, which are not the peaks of an QRS complex, are sorted out and omitted by this procedure. On the other hand, in an ECG signal with ventricular fibrillation only such peaks are preserved, which are peaks of a fibrillation period.

In other words: Most of the relative maxima, which exist due to noise in the ECG signal are deleted. Furthermore, nearly all relative maxima, which are peaks of insignificant elevations (in this algorithm also P waves and T waves) are deleted as well. This selection procedure is carried out by setting adaptive thresholds. The value of the thresholds is calculated by use of different parameters, that were selected by experiments with ECG signals. The result is a set of points *X*, which is a subset of *M*_0. _In fact, the temporal appearance of the points in *X *is related to the frequency of the heart beat. The average frequency found by this points is related to a certain probability factor. This factor, together with other results, is finally used to make a decision whether the signal is VF or not.

Now, the central idea of the algorithm is applied. The points in *X *are used to generate four artificial signals. The first signal looks like a normal sinus rhythm, that has its QRS peaks exactly at the points of *X*. A reference signal which consists of one PQRST segment is fitted from one maximum of *X *to the next. To fit the different size of the peaks it is scaled linearly. It has all features that a normal ECG signal should have (narrow QRS complex, P wave, T wave). The second artificial signal is the average of about 700 normal sinus rhythm signals found in 16 files of the MIT database and 16 files of the CU database. The third artificial signal has QRS complexes and an elevated T wave. The fourth signal, which we use as a reference for a ventricular fibrillation signal, has the shape of a cosine like function, which has its peaks at the points of *X *and therefore simulates ventricular fibrillation.

The next step is the calculation of the residuals with respect to the reference signals. We call the ECG signal *E*(*t*), the reference signals that simulate a healthy heart *S*_*j*_(*t*), *j *= 1,2,3, and the ventricular fibrillation signal *F*(*t*). The following parameters are calculated



where *I *= [*t*_0_, *t*_1_] with *t*_0 _= min {*t*_*j*_|*t*_*j *_∊ *X*} and *t*_1 _= max {*t*_*j*_|*t*_*j *_∊ *X*}. Thus, *I *is the temporal interval from the smallest *t*_*j *_in *X *to the largest *t*_*j *_in *X*. Now, four further values are calculated



*c*_1 _and *c*_2 _are two constants that were suitably chosen by tests. Finally, *VRF *and *VRS *are compared. If all *t*_*j*_, *j *= 1, 2, 3 are smaller than 1, the signal is classified as VF, otherwise it is considered to be SR. (Note 6: Using an *L*^2 ^norm did not improve the quality.)

The critical threshold parameter to obtain the ROC are *t*_*j*,0_.

### Wavelet based algorithms

The continuous wavelet transform of a signal *f *∊ *L*^2 ^is defined by



where *ψ *is the mother wavelet, *ψ *∊ *L*^2^, and admissible, i.e.,



Here,  denotes the Fourier transform of *ψ*



The wavelet transform *L*_*ψ*_*f *contains information about the frequency distribution as well as information on the time distribution of a signal.

According to Lemma 1.1.7 from [15], the Fourier transform of *L*_*ψ*_*f *is given by



#### WVL_1_

A new simple wavelet based algorithms (WVL_1_) operates like SPEC in the frequency domain. The idea of this first wavelet algorithm is the following: First, a continuous wavelet transform of the ECG signal is carried out using a Mexican hat as mother wavelet. Then a Fourier transform is performed. Now, the maximum absolute values are investigated in order to make the decisions for the defibrillation process. However, one can show that these maximum values are located on a hyperbola in the (*a*, *w*) plane of the Fourier transform of the wavelet transform of the ECG signal, i.e., on a curve that has the representation *aw *= *C*, *C *being a constant. The values on this curve in the (*a*, *w*) plane are the Fourier transform of the ECG signal multiplied by a weight function *g*(*w*). Therefore, if one searches for the maximum values of  in the (*a*, *w*) plane of the wavelet transform, it is sufficient to search for the maxima of the weighted Fourier transform of the ECG signal .

Since we are looking for maxima of the modulus of , we need to consider the maxima of  only. In WVL_1 _the function  is handled exactly like the spectrum in the algorithm SPEC. The same spectrum parameters are calculated and also the thresholds for the decision have the same values like the algorithm in SPEC.

In WVL_1_, the critical threshold parameter to obtain the ROC is *A*_2,0_.

#### WVL_2_

This new method of detecting ventricular fibrillation uses a discrete wavelet transform. It is split into two parts.

##### (i) Finding VF

The first part uses the algorithm SPEC to search for typical VF properties in the ECG. If the algorithm decides that the ECG part contains VF, then the result is accepted as true and no further investigation is carried out. This procedure can be justified by the high specificity of the SPEC algorithm. If the algorithm yields that the ECG part is "no VF", a further investigation is carried out to confirm this result or to disprove it.

##### (ii) Discrete Wavelet Transform (DWT)

This part is only carried out, if the first part of the algorithm considers the ECG episode to be "no VF". In this case a discrete wavelet transform is applied, that searches for QRS complexes in the following way:

The third scale of a discrete wavelet transform with 12 scales and a "Daubechies8" wavelet family is used. Numerical tests have shown that this scale makes it easiest to distinguish VF from "no VF". If the signal in the third scale has a value higher than a certain threshold, the according ECG part is considered as QRS complex. The threshold used in this investigation is set to 0.14 max(ECG signal). Multiple peaks belonging to the same QRS complex are removed.

If more than two but less than 40 QRS complexes are found within an 8 second episode, "no VF" is diagnosed. Otherwise the two spectral parameters *FSMN *and *A*_2 _from the first part are investigated again. If *FSMN *< 2.5 and *A*_2 _> 0.2, the considered ECG part is diagnosed as VF.

The mentioned range for the number of found QRS complexes has the following reason: Sometimes, especially in ECGs with a high amount of noise, the DWT part makes wrong interpretations and "finds" QRS complexes also in QRS free episodes. Therefore, a minimal number of three QRS complexes is demanded to confirm the existence of QRS complexes. On the other side, if the DWT part "finds" more than 40 QRS complexes (equal to a pulse of 300 beats per minute), the signal is likely to be VF, since such high sinus rhythms do not appear. The limits of the range were chosen from experiments with data.

In WVL_2 _no IROC is calculated due to the special structure of the algorithm. Since it consists of two parts and the second part is not executed always, we do not have a single parameter that includes the calculations of both algorithm parts in every ECG segment. Hence we cannot calculate an IROC value. Using the parameters of the SPEC algorithm as an IROC parameter does not yield an ROC curve over the full range.

*Finally we want to compare the VF detection algorithms with two algorithms, that are originally used for QRS detection*. The decision thresholds of these algorithms have been optimized to be suitable for VF detection.

### Li algorithm

The Li algorithm (LI) [16] is based on wavelet analysis, too.

The wavelet transform of an ECG signal is calculated using the following equations





Here,  is a smoothing operator and  being the ECG signal. *h*_*k *_and *g*_*k *_are coefficients of a lowpass filter *H*(*w*) and a highpass filter *G*(*w*), respectively. Scales 2^1 ^to 2^4 ^are selected to carry out the search for QRS complexes. QRS complexes are found by comparing energies from the ECG signal in the scale 2^3 ^with the energies in the scale 2^4^. Redundant modulus maximum lines are eliminated and the R peaks detected. Different methods from [17] are used to improve the detection quality:

#### Method 1

Blanking, where events immediately following a QRS detection are ignored for a period of 200 *ms*.

#### Method 2

Searching back, where previously rejected events are reevaluated when a significant time has passed without finding a QRS complex. If no QRS complex was detected within 150% of the latest average RR interval, then the modulus maxima are detected again at scale 2^3 ^with a new threshold.

If the number of found QRS complexes is 0 or higher than 5 times the window length in seconds, the ECG segment is classified as VF.

The critical threshold parameter to obtain the ROC is the number of found QRS complexes.

### Tompkins algorithm

This algorithm is based on a QRS complex search (TOMP) [18]. It uses slope, amplitude and width information to carry out this task.

After preprocessing, the ECG signal is band filtered by a low pass filter and a high pass filter to reduce interference and high frequency noise. Then, the signal is differentiated to provide the QRS complex slope information. The difference equation for the slope *y*(*j*) of the ECG data *x*(*j*) reads



where *T *is the sampling period of the ECG signal. Afterwards the signal is squared to make all data points positive. A moving window integration with a window width of 150 *ms *(e.g., 54 points at a sampling rate of 360 *Hz*) is applied. Thresholds are set up to detect QRS complexes.

This algorithm uses a dual threshold technique and a searchback for missed beats. If the number of found QRS complexes is smaller than *l*_0 _= 2 or higher than *l*_1 _= 32, the ECG segment is classified as VF.

The critical threshold parameter to obtain the ROC is *l*_0._

## 2 Results

For all algorithms tested in this paper we used the same prefiltering process described at the beginning of the previous section. The function *filtering.m *for preprocessing can be found on the website .

First, we investigated ECG episodes of window length according to the original papers, and then of window length of 8 seconds since that yielded the best results. For the investigation we simulated a continuous analysis by selecting the data in steps of one second. The decision of an algorithm analyzing an episode of a certain window length was assigned to the endpoint of that interval. By its very nature this continuous monitoring of an ECG signal contains transitions of different rhythms. All algorithms were tested under equal conditions. Finally, we recorded the results together with the annotations in an output file.

The quality parameters are presented in the following tables and figures. The perfect algorithm would have values for sensitivity, specificity, positive predictivity, accuracy and IROC of 100%, assuming that the annotations are 100% correct.

The data sets were taken from the BIH-MIT database (48 files, 2 channels per file, each channel 1805 seconds long), the CU database (35 files, 1 channel per file, each channel 508 seconds long), and the AHA database (files 7001 – 8210, 40 files, 2 channels per file, each channel 1800 seconds long).

Note 7: ANSI/AAMI EC38:1998 Ambulatory electrocardiographs: "The incidence and variety of VF in the AHA and MIT databases are not sufficient to allow those databases to serve as substitutes for the CU DB for the purposes of 5.2.14.5. An evaluation of VF detection using the 80 records of the AHA DB and the 48 records of the MIT DB should supplement the required CU DB evaluation, however, as the CU DB does not contain a sufficient sample of signals likely to provoke false VF detections."

Thus, the total number of decisions per algorithm (window length = 8*s*) was 2·48·(1805 - 7) + 35·(508 - 7) + 2·40·(1800 - 7) = 333 583.

The annotations of these databases are on a beat to beat level. When taking an arbitrary 8 second episode which includes a VF sequence at the end, it was assumed that the overall classification is VF.

The testing was done automatically by an application written with MATLAB, since there is no chance to inspect 330 000 ECG episodes by hand.

### Numerical results

Table 1 shows the values for the sensitivity, the specificity and the integrated receiver operating characteristic of the investigated algorithms. The great differences in performance on different databases can be easily explained by the different nature of this databases (see Note 7). The overall results were directly calculated from the 333 583 decisions of VF/no VF.

Table 2 shows the values for the positive predictivity, the accuracy and the calculation time of the investigated algorithms.

Table 3 shows the values for the sensitivity of the investigated algorithms, if, due to an appropriate adaption of the threshold parameters, the specificity were 95% and 99%, respectively.

Figure 5 and 6 show the ROC curves for all algorithms.

For the computation of the ROC curves, we used 64 nodes. Since some critical threshold parameters are discrete the points of the ROC curve are not equidistant.

## 3 Discussion and Conclusion

In real applications of AEDs the specificity is more important than the sensitivity, since no patient should be defibrillated due to an analysis error which might cause cardiac arrest. Therefore, a low number of false positive decisions should be achieved, even if this process makes the number of false negative decisions higher. But one has to distinguish between our calculated values for specificity and sensitivity and the values in [19]. Our values were determined for the basic mathematical algorithms, whereas this paper gives recommendations for whole ECG analysis systems. It also does not consider an analysis without preselection.

Our results show that no algorithm achieves its proclaimed values for the sensitivity or specificity as described in the original papers or in [5] and [6] when applied to an arbitrary ECG episode. The main reason for this is the following: Whereas all other researchers made a preselection of signals, we simulated the situation of a bystander, who is supposed to use an AED, more accurately. Hence no preselection of ECG episodes were made.

The best algorithm SCA, which yields the best value for the integrated receiver operating characteristic (IROC) is a new algorithm followed by the algorithms SPEC and VF. Studying the ROC curves in Figure 5 and Figure 6 we see that the relevant part of the ROC curves lies at the left side. The ROC curve also enables us to compare different algorithms given a specified specificity.

All other algorithms yielded only mixed results in our simulations. We also conclude that algorithms developed for QRS detection, like LI and TOMP, are not suitable for VF detection even when the thresholds are suitably adapted.

### Outlook

The currently best algorithm works in the time domain. The two algorithms SPEC and VF use information on the energy distribution from the frequency domain but do not use any corresponding phase information. Whereas the algorithm CPLX which uses methods from chaos theory has a poor performance in the region where Specificity > 80% our current investigations indicate a promising good performance for new algorithms based on other methods coming from chaos theory which are currently under development. When finished these algorithms will be presented elsewhere.

## Appendix A: Sensitivity, Specificity, Positive Predictivity, Accuracy, and ROC

Sensitivity is the ability (probability) to detect ventricular fibrillation. It is given by the quotient



with *TP *being the number of true positive decisions, *FN *the number of false negative decisions. Specificity is the probability to identify "no VF" correctly.

It is given by the quotient



where *TN *is the number of true negative decisions, and *FP *is the number of false positive decisions. This means that if a defibrillator has a sensitivity of 90% and a specificity of 99%, it is able 90% of the time to detect a rhythm that should be defibrillated, and 99% of the time to recommend not shocking when defibrillation is not indicated.

Remark: a trivial algorithm which classifies every ECG episode as "no VF" will reach a specificity of 100%, but will have sensitivity 0%. On the other hand, a trivial algorithm which classifies every ECG episode as VF will reach a sensitivity of 100%, but will have specificity 0%. The ROC curve (see below) describes this inherent tradeoff between sensitivity and specificity.

Furthermore, we calculated the *Positive Predictivity *and the *Accuracy *of the investigated algorithms.

Positive predictivity is defined by



Positive predictivity is the probability, that classified VF is truly VF:

Accuracy is defined by



Accuracy is the probability to obtain a correct decision.

Specificity and sensitivity always depend on the chosen critical threshold parameters which depend, on the other hand, on the databases used for evaluation (see Note 7).

To get rid of at least of the dependence on the chosen critical threshold parameter one uses the ROC curve. The sensitivity is plotted in dependence of (1 - specificity), where different points in the plot are obtained by varying the critical threshold parameter in the decision stage of an algorithm.

The ROC curves enables us to compare different algorithms when choosing a specified specificity. For more information on ROC curves see [20,21].

## Appendix B: Algorithm details: Signal Comparison Algorithm (SCA)

Here we describe the search for relative maxima and the appropriate choice among them, used in Section 1 in more detail.

An offset is added to the ECG signal to make its mean value to zero. We construct a set *Z *containing the values *a*_*j *_and temporal positions *t*_*j *_of this new signal, i.e., *Z *= {(*t*_*j*_, *a*_*j*_)|*a*_*j *_is the value of the ECG signal at time *t*_*j*_}.

All further steps are executed both with the set *Z *and the set -*Z*, where -*Z *= {(*t*_*j*_, *b*_*j*_)|*b*_*j *_= -*a*_*j *_is the value of the negative ECG signal at time *t*_*j*_} with the help of the reference signals *rECG*_ℓ_, ℓ being *VF*, *SR*_1_, *SR*_2 _or *SR*_3_, or, equivalently, ℓ = 0,1, 2, 3. Note, that the maxima of *Z *correspond to the minima of -*Z*. So we get 2 * 4 = 8 tests to find out whether a signal is VF or SR. If any of the 8 tests yields SR, the signal is considered to be SR.

### Step 1

**Figure 7:**
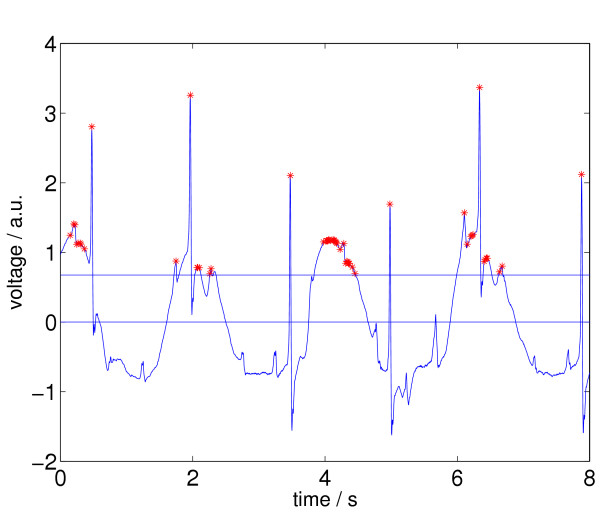
ECG signal with relative maxima (indicated by stars left) after applying step 1 of Signal Comparison Algorithm (SCA). This ECG episode is annotated as no VF, a.u. ... arbitrary units.

All relative maxima *a*_*j *_of *Z *and their corresponding times *t*_*j *_are determined. The resulting set is called *M*_0_, i.e., *M*_0 _= {(*t*_*j*_, *a*_*j*_)|*a*_*j *_is a local maximum}, so *M*_0 _⊂ *Z*. All *a*_*j *_in *M*_0_, that are smaller than *A*, where *A *= Δ·max(*a*_*j*_) and Δ is a threshold, are deleted. The threshold Δ is set to 0.1 for the VF reference signal and to 0.2 for the SR reference signals. We call the reduced set *M*_1_. In Figure 7 we see an ECG episode from the CU database (cu21, from *t *= 148 *s *until *t *= 156 *s*) together with its selected relative maxima according to the status after processing step 1.

Now, we introduce an index *l *and set it to *l *= 1.

### Step 2

*M*_*l *_is reduced further: The maximum *a*_*j *_in *M*_*l *_is searched. Here, we call it *a*_*max*_. *a*_*max *_has a corresponding temporal position *t*_*max*_. Then, the largest possible temporal interval *I*_*l *_in *Z *around *t*_*max *_is searched, so that all values *a*_*j *_in this interval are equal or smaller than *a*_*max *_and larger than 0.2*a*_*max*_. All pairs (*a*_*j*_, *t*_*j*_) except (*a*_*max*_, *t*_*max*_) in *M*_*l*_, that are referred to the found interval *I*_*l*_, are deleted. We get a set that we call *M*_*l*+1_. This procedure is repeated with all untreated *a*_*j *_in *M*_*l*_, until every *a*_*j *_has been considered and afterwards either been deleted or kept. After each step, *l *is increased by 1. This means, first we consider *M*_1_, then *M*_2 _= *M*_1_\*I*_1_, then *M*_3 _= *M*_1_\{*I*_1 _∪ *I*_2_} and so on, until we reach a highest *l*, called *l*_*max*_. In the end, we get a set that we call *M*, with .

**Figure 8:**
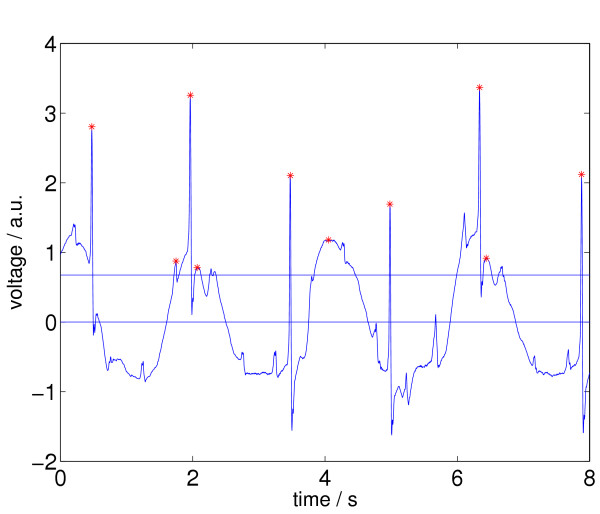
ECG signal with relative maxima left (indicated by stars) after applying step 2 of Signal Comparison Algorithm (SCA).

In the end, the *a*_*j *_in *M *are the relative maxima in *Z*, that are higher than *A *and are the only ones in certain subintervals of *Z*. Two different *a*_*j *_in *M *can only be neighbors in *Z*, if they are separated by a valley that is deeper than 20% of the higher peak of the two. In Figure 8 we again see the ECG episode, together with its newly selected relative maxima according to the status after processing step 2.

### Step 3

A value Ω is calculated from M. The frequency Ω of "peaks" is given by



where *N*_*M *_is the number of points in *M *and *t*_*max *_- *t*_*min *_is the maximum temporal range of the elements in *M*.

### Step 4

**Figure 9:**
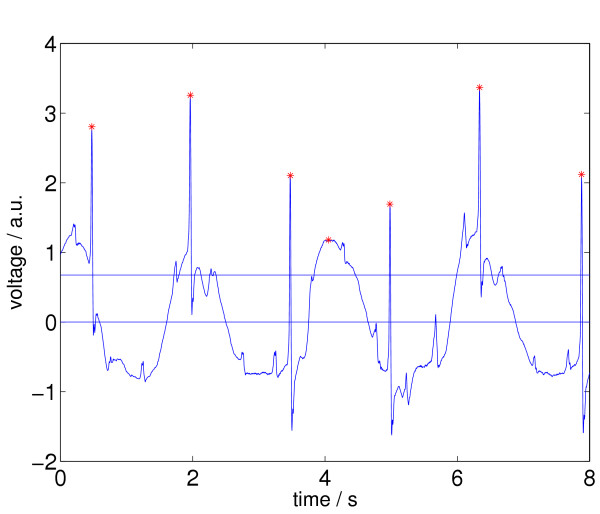
ECG signal with relative maxima (indicated by stars) after applying step 4 of Signal Comparison Algorithm (SCA).

Now, if two different elements (*a*_*i*_, *t*_*i*_) and (*a*_*j*_, *t*_*j*_) of *M *are separated by a temporal distance |*t*_*i *_- *t*_*j*_| smaller than , the element with the smaller *a *is deleted from *M*. This final set is called *X*. In Figure 9 we again see the ECG episode as in the figures above and together with its newly selected relative maxima according to the status after processing step 4.

### Step 5

Ω is recalculated by Equation (28) with the help of the recalculated set *X*. If Ω > 280, *r *is set to 2, if Ω < 180, *r *is set to 0.9, else *r *is set to 1.

### Step 6

The decision is calculated by Equation (16). *VRF *is calculated for the ventricular fibrillation reference signal, *VRS *for the sinus rhythm reference signal.

**Figure 10:**
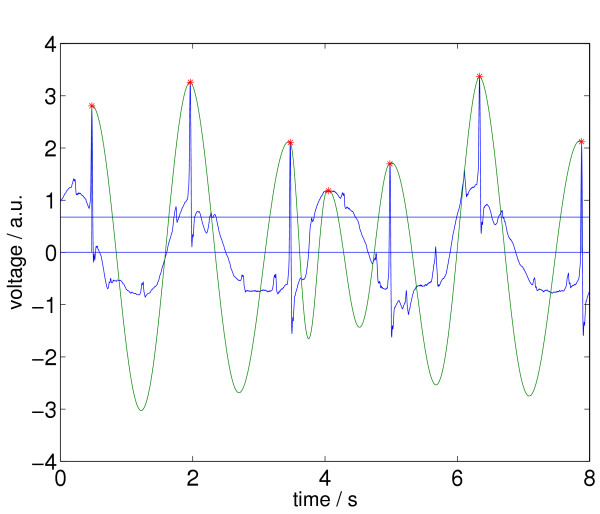
ECG signal with relative maxima and VF reference signal after applying step 6 of Signal Comparison Algorithm (SCA).

In Figure 10 we see the ECG episode together with the corresponding VF reference signal.

**Figure 11:**
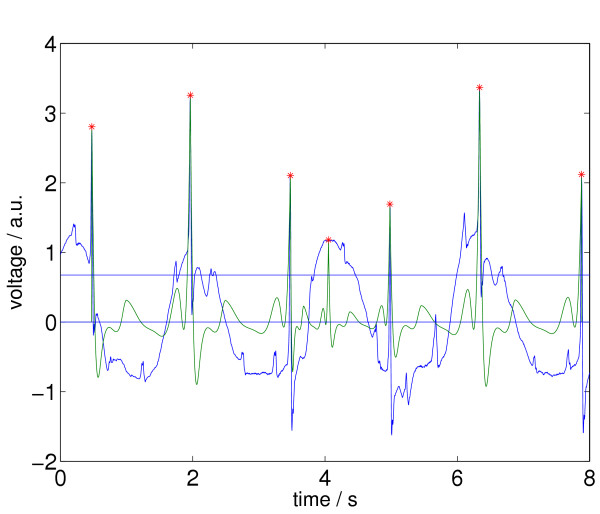
ECG signal with relative maxima and SR reference signal after applying step 6 of Signal Comparison Algorithm (SCA).

In Figure 11 we see the ECG episode together with the first corresponding SR reference signal. *c*_1 _is set to 2/*r*, *c*_2 _is set to 1.
